# Chitosan/polyethylene glycol fumarate blend films for wound dressing application: in vitro biocompatibility and biodegradability assays

**DOI:** 10.1007/s40204-018-0093-2

**Published:** 2018-07-18

**Authors:** Azadehsadat Hashemi Doulabi, Hamid Mirzadeh, Mohammad Imani, Shadab Bagheri-Khoulenjani

**Affiliations:** 1 Department of Polymer Engineering, Amirkabir University of Technology, P.O. Box 15875/4413, Tehran, Iran; 2 Department of Novel Drug Delivery Systems, Iran Polymer and Petrochemical Institute, P.O. Box 14965/115, Tehran, Iran

**Keywords:** Chitosan, Blend, Biodegradation, Cell behaviour, Wound dressing

## Abstract

Blending is one of the effective approaches in preparing tailored materials with a wide range of properties. Thus, chitosan-based polymers have been fabricated and used as wound dressings since they possess better properties than those of the constituent materials. The objective of this work was to evaluate the biocompatibility and biodegradability of biodegradable blend films based on polyethylene glycol-*co*-fumarate (PEGF) and chitosan (Ch). The blend films of Ch/PEGF were prepared by solution casting/solvent evaporation method. Degradation behavior of these blend films was evaluated in a simulated fluid at physiological pH supplemented with lysozyme at a concentration similar to that in human serum by weight loss of the films and changes in the pH of media. When the pH of incubation media was analyzed, with an increase of PEGF content in the blend films, the degradation rate increased accordingly. The pH of the media of samples was not significantly changed at any measured time point and all films kept their integrities during 28 days. The biocompatibility of the films and cell behavior on the surface of these films were investigated by in vitro tests. Biological assessment using mouse fibroblast cell line L929 on the blend films of Ch/PEGF indicated that films supported the attachment, spreading and proliferation of cells. Since the Ch/PEGF films are biocompatible with the tailored biodegradation rate, they might have a great prospective position in the application of wound dressings.

## Introduction

The degradation rate of a wound dressing film has to match the rate of wound healing during the healing process (Cao and Wang 2009). Faster degradation is not desirable as the healing will remain unsupported and a slower degradation rate might cause an unwanted immune reaction or host response. Hence, a trade-off has to be achieved between structural integrity and the rate of degradation of the wound covering polymeric mass (Kean and Thanou 2010). Chitosan (Ch) is a biocompatible, biodegradable, non-toxic and antibacterial biopolymer that has been well known as being able to accelerate the healing process of wounds in humans (Mir et al. 2018; Muzzarelli 2009). Blending is one of the effective approaches to prepare a material as tailored and with a wide range of properties (Ahmed and Ikram 2016; Muxika et al. 2017; Usman et al. 2016). Poly(ethylene glycol-*co*-fumarate) (PEGF) block copolymer is a biocompatible, cytocompatible, biodegradable aliphatic unsaturated polyester with tunable mechanical properties (Hashemi Doulabi et al. 2013a). The Ch/PEGF blend films were prepared for obviating the inherent drawbacks of neat Ch and neat PEGF for wound dressing applications and simultaneously improving Ch/PEGF biological properties (Hashemi Doulabi et al. 2013a, b). Chitosan degrades into oligosaccharides by the enzymatic action of lysozyme in vivo (Nordtveit et al. 1996) and PEGF undergoes non-enzymatic bulk hydrolysis of its ester linkage followed by fragmentation into oligomers (Hashemi Doulabi et al. 2008). Preparing a blend film of these two polymers in different blend ratios seems to tailor the surface properties such as surface roughness resulting in changes of cell adhesion. It was reported that Ch/PEGF blend films were potential candidates as wound dressings due to some of their properties such as antibacterial activity, permeability and fluid absorption (Hashemi Doulabi et al. 2013b, 2015). Motivated by our preliminary results (Hashemi Doulabi et al. 2013b), the main goal of this research was to investigate in vitro degradability, biocompatibility and cytocompatibility of Ch/PEGF blend films as important requirements for a wound dressing material. Their biodegradation profile and in vitro cell behavior response confirmed their potential for medical applications. To the best of our knowledge, long-term in vitro degradation studies of the Ch/PEGF blend film at simulated physiological conditions, i.e., pH, enzyme, etc., as well as cell behavior evaluations have not yet been published.

## Materials and methods

### Materials

Low-viscosity Ch (~ 80% DD; 20–200 mPa s, Fluka, Germany) was purified as reported elsewhere (Hashemi Doulabi et al. 2013c). Hen egg white (HEW) lysozyme (46,400 U/mg) and glutaraldehyde were purchased from Sigma-Aldrich (Germany). PEGF (*M*_w_: 19, *M*_*n*_: 16 kDa) was synthesized in-house previously by condensation polymerization of fumaryl chloride with polyethylene glycol (PEG) diol *M*_w_ = 3 kDa, Merck Chemicals, Dusseldorf, Germany) in the presence of propylene oxide (Hashemi Doulabi et al. 2008). Fumaryl chloride and propylene oxide were obtained from Aldrich, Milwaukee, MN, USA. All other chemicals used were of reagent grade.

### Blend film preparation

Chitosan was blended with PEGF in different blend ratios (40/60, 60/40, 80/20) and processed into films as previously described (Hashemi Doulabi et al. 2013a). Briefly, filtered mixtures of Ch and PEGF containing 1 g/dL of solid content were dissolved in 1% v/v aqueous acetic acid and poured into petri dishes and left to completely dry at room temperature and then dried in vacuo for 48 h.

### Biodegradation assay

In vitro degradation profile of blend films (1.5 cm × 1.5 cm) with average thickness of 30–70 μm was followed for a duration of 4 weeks in PBS containing 1.5 μg/mL HEW lysozyme. The test was performed in accordance with the procedure described elsewhere with some modifications (Li et al. 2010). Briefly, films with calculated dry weight were neutralized in 1 N NaOH, washed with deionized water and incubated in the lysozyme solution with gentle mechanical agitation at 37.5 °C. The samples were removed from the medium at predetermined time intervals, i.e., 1, 7, 14 and 28 days and the pH of the incubation media was also recorded. The samples were washed with deionized water, dehydrated using absolute ethanol and dried to constant weight in an oven (40 °C) and weighted. The weight loss (%) at different intervals was calculated by Eq. (1) (Tanuma et al. 2010). To separate between enzymatic degradation and dissolution, control samples were incubated for 28 days under the same conditions (PBS, *T* = 37.5 °C and pH 7.4) without the enzyme addition. Media were replaced every 2 days.

1 $${\text{Weight loss }}(\% ) = \frac{{W_{0} - W_{t} }}{{W_{0} }} \times 100,$$

where *W*_0_ is the initial weight before degradation test and *W*_*t*_ is the dry weight at predetermined time *t*.

### In vitro study

Biocompatibility and cytocompatibility tests were carried out by exposing a cell line of mouse fibroblast (L929) with the films after one, three and five incubation days, in vitro. These assays were accomplished according to the procedures reported by Luna et al. (2011). Cultured mouse fibroblasts (L929, the cell bank of National Institute of Genetic Engineering and Biotechnology, Iran) were harvested with a 0.25% Trypsin–EDTA solution (Sigma) in a phosphate-buffered saline (PBS; pH 7.4) and re-suspended in the culture medium composed of RPMI-1640 with l-glutamine (Sigma, USA), supplemented with 10% fetal bovine serum (FBS, Gibco, Germany), 1% Pen-Strep (100 IU/mL penicillin and 10 mg/mL streptomycin, Sigma-Aldrich, USA). A cell suspension was prepared with a concentration of approximately 1 × 10^6^ cell/mL and seeded onto 12-well plates in contact with the sterilized films. These plates were incubated for 1, 3 and 5 days at 37 °C in a wet atmosphere containing 5% CO_2_. Afterward, the images of cells fixed on the film surfaces were taken by scanning electron microscopy (SEM). To this end, cells were fixed using 2.5% glutaraldehyde (Sigma-Aldrich, Germany) in a PBS solution and dehydrated using a series of ethanol solutions (25, 30, 50, 70, 80, 90, 100% v/v). The samples were dried overnight at room temperature, coated with gold by sputtering and observed by SEM. Cell count and cell spreading measurements were conducted by Image-pro Plus 6 software (Version 6.0.0. 260, Media Cybernetics Inc.). Cell count and cell spreading measurements were conducted using Image-pro Plus 6 software (Version 6.0.0. 260, Media Cybernetics Inc.) (Karkhaneh et al. 2011).

The cell viability was evaluated by MTT 3-(4,5-dimethylthiazol-2-yl)-2,5-diphenyltetrazolium bromide, Merck, Germany) assay by enzymatic conversion of MTT after the time intervals of 1, 3 and 5 days and the results were expressed as a percentage of cell viability. A cell suspension was prepared with a concentration of approximately 6.2 × 10^5^ cell/mL and seeded onto 96-well plates containing sterilized films. After each interval, 100 μL of MTT solution (0.5 mg/mL in PBS) was added to each well and incubated for 4 h at 37 °C in a humidified atmosphere. The MTT reagent was then removed from the wells and 200 μL 2-propanol was added to dissolve the formazan crystals. Cell viability has been determined using Absorbance Microplate Reader (BioTek ELx808, USA) at 570 nm with the reference wavelength of 650 nm.

### Statistical analyses

All experiments were repeated three times with triplicate samples for each group. Analysis of variance (ANOVA) and linear regression were the main statistical tools used for data analysis. The Tukey (*α* = 0.05, 95% confidence intervals) was also used to determine the significance of differences observed between specific means (Origin^®^, 7.0, 2002, USA).

## Results and discussion

### Biodegradation assay

**Fig. 1:**
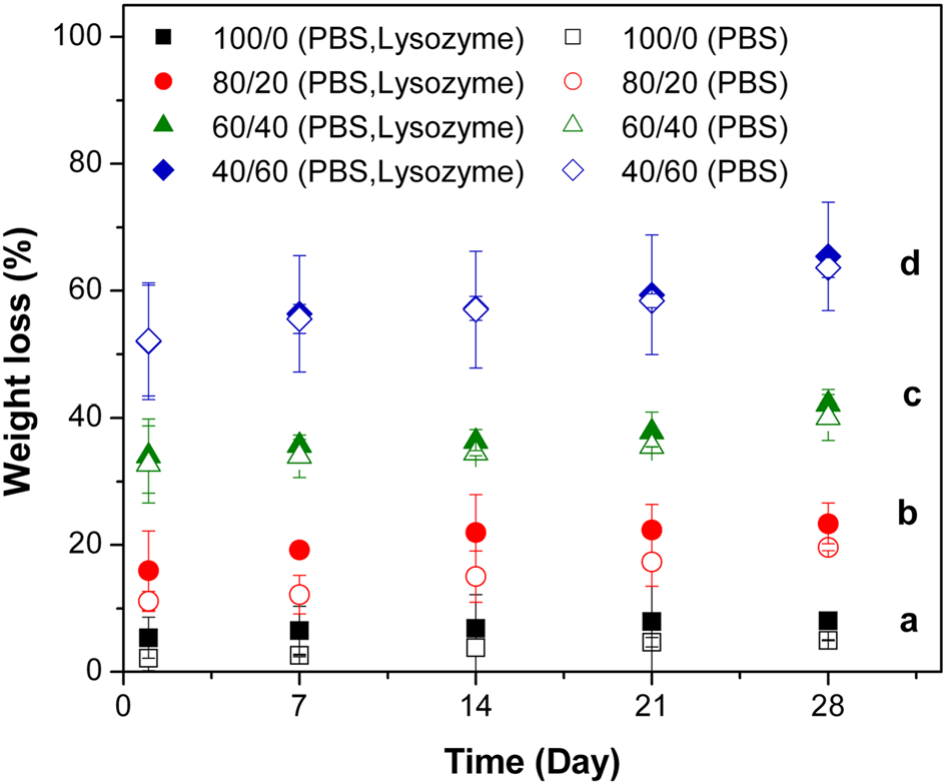
In vitro degradation behaviors of Ch and Ch/PEGF blend films with different blend ratios: weight loss vs. the time in two types of media—with/without lysozyme

Chitosan is enzymatically degradable in human serum in the presence of lysozyme by hydrolyzing glycosidic bonds present in the Ch backbone (Nordtveit et al. 1996). In this study, biodegradation behavior was characterized at physiological pH and enzyme concentration similar to that in human serum. Figure 1 shows an increment in the weight loss of the blend films with increasing the PEGF content in the composition of the films (*p *< 0.05). On day 1 of incubation, the film with 80/20 in the blend ratio showed the least dissolution among the blends, degradation in media without enzyme (ca. 11%) according to weight loss results due to PEGF content in its formulation. Enzymatic degradation and simple dissolution of Ch/PEGF blend films with different blend ratios were compared together. As seen in Fig. 1, it seems that the weight loss of the film with 80/20 in blend ratio in the enzyme-supplemented medium was greater than the one in PBS without lysozyme due to the loss of Ch. In contrast to this blend, the effect of lysozyme on degradation rates of the films with 60/40 and 40/60 in blend ratios between two groups, the enzyme-supplemented medium and PBS without lysozyme was not statistically significant (*p *> 0.05). In other words, Ch degradation was not effectively influenced by the enzyme concentration here. It may be attributed to the highly deacetylated Ch which is less susceptible to lysozyme (Nordtveit et al. 1996; VandeVord et al. 2002). Moreover, as the PEGF content in the blend films increased, the weight loss differences between the two groups were lost due to the prevalent contribution of hydrolytic degradation of PEGF segments; thus the hydrolytic degradation phenomenon could cover the enzymatic degradation outcome. Therefore, the effect of lysozyme was negligible within 28 days and for enzymatic degradation.

**Fig. 2:**
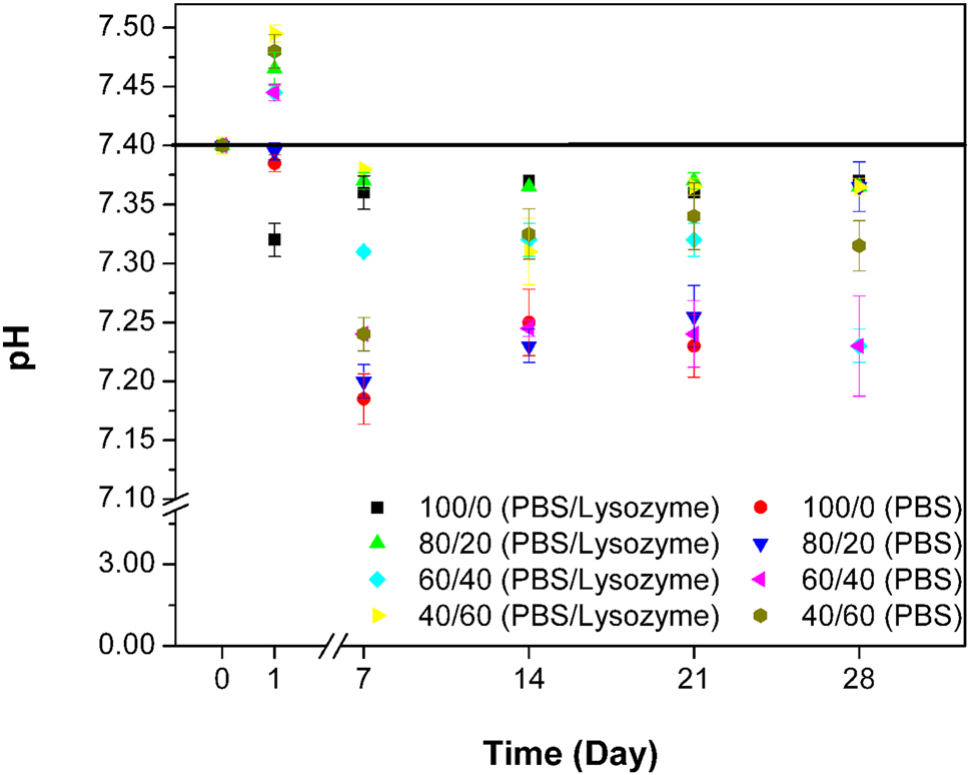
In vitro degradation behaviors of Ch and Ch/PEGF blend films with different blend ratios: pH changes vs. the time in two types of media—with/without lysozyme

When a medium contains Ch, the pH of the environment could influence the patterns of solubility and enzymatic degradation of Ch. It is worthy to mention that Ch is soluble in aqueous solution only at pH < 6.2, while its degradation rate reaches the maximum value at a pH of 5.2. To clarify the degradation behavior of the Ch-based polymer, the pH of incubation media was recorded and analyzed (Fig. 2). Interestingly, on the first day, there was a statistically significant increase in the pH of all sample media from 7.44 to 7.52 (*p *< 0.05). This may be attributed to semi-stable adherence of NaOH, to Ch through the naturalizing process as reported for the blend of Ch and PCL which has also reported by Sarasam et al. (2006). Figure 2 shows that the pH changes in the polymer degradation media due to different film compositions were not significantly different at any measured time point and the pH was nearly set on an average value of 7.35 throughout the experiment. The free PEG segments presented in the sol fraction also did not influence the pH. Another important point was the blend films with 80/20, 60/40 in Ch/PEGF that keep their integrities and shapes well after 28 days of tests, which means these films can cover the wound area during the healing process. Therefore, they are suitable for application as wound dressings.

### Cell morphology

**Fig. 3:**
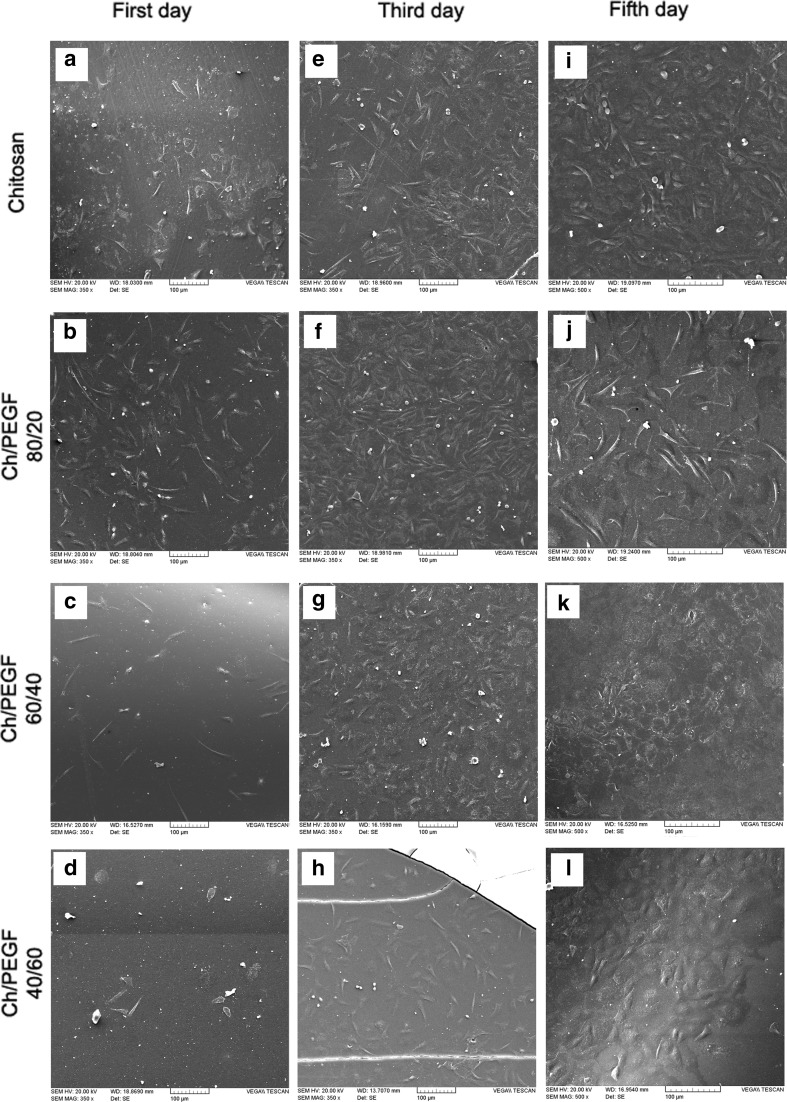
SEM micrographs of L929 fibroblast cell behaviors in contact with Ch and Ch/PEGF blend films after different incubation intervals as shown on the images (**a**–**l**), scale bar 100 μm

**Fig. 4:**
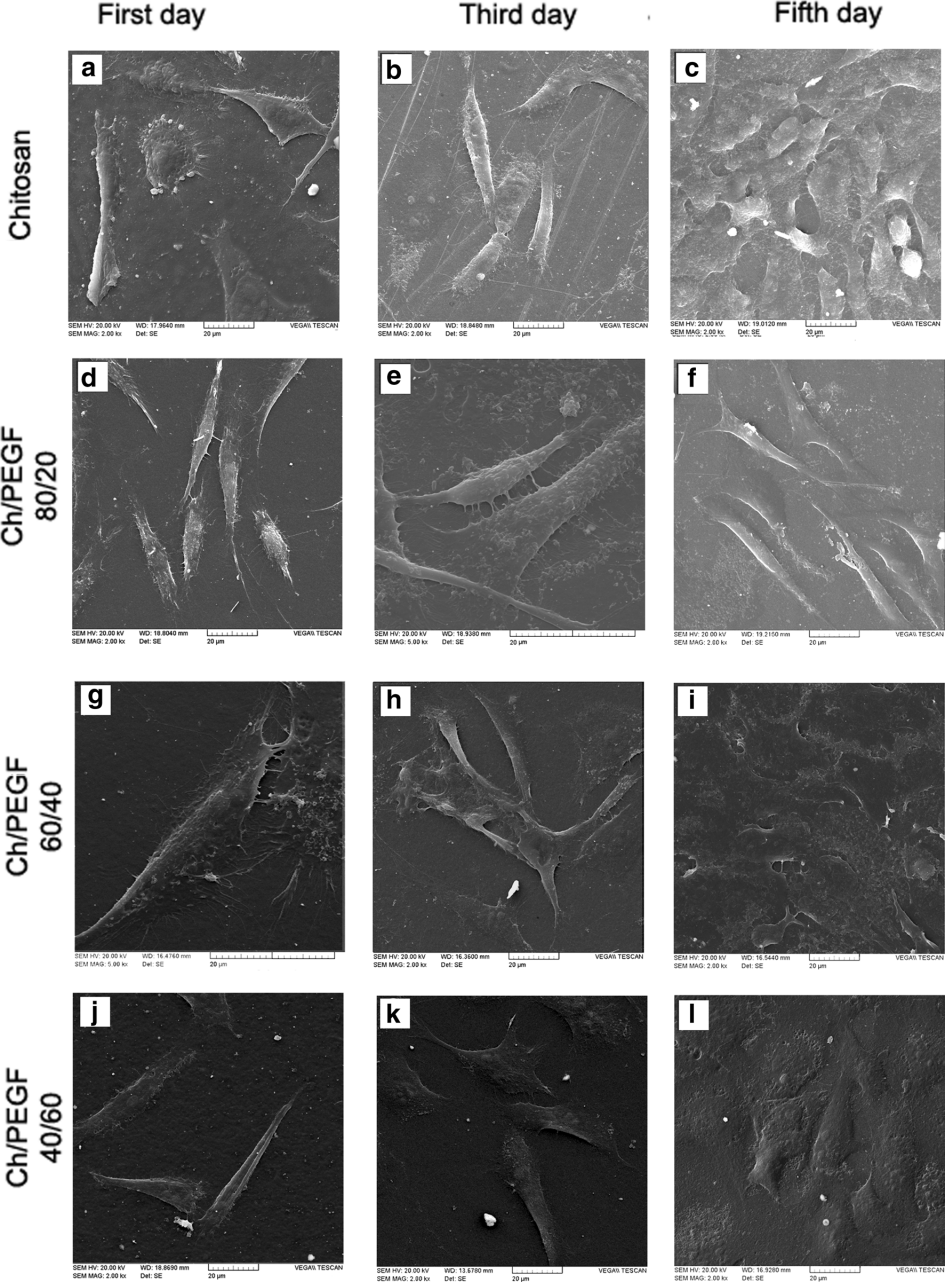
SEM micrographs of L929 fibroblast cell behaviors in contact with Ch and Ch/PEGF blend films after different incubation intervals as shown in the images (**a**–**l**), scale bar 20 μm

It has been reported that the properties of the blend surface could influence the cell attachment (Zhang et al. 2002). The cellular behavior in response to a biomaterial is an important factor determining the biocompatibility. When cells contact the biomaterial, cells undergo different morphological changes to stabilize the cell–material interface (Wang et al. 2003). The whole process of adhesion and spreading of cells on the biomaterial surface includes four factors cell attachment, filopodial growth, cytoplasmic webbing, and flattening of the cell mass. Cell adhesion depends on several factors such as surface topography, surface chemistry, surface energy, and hydrophilicity as well as sample chemical composition (Hashemi Doulabi et al. 2013b). In other words, the presence of some effective cell/surface interactions, suitable wettability, and adequate roughness are some examples that could improve cell affinity. Figure 3a–l shows cell adhesions and morphologies of L929 fibroblasts cultured on the Ch and its blend films after 1, 3 and 5 days of incubation, indicating that they are alive on the hydrogels and suggesting that our hydrogel is biocompatible in vitro. It can be seen that the cultured cells on the first day show clear morphologies in different stages (Fig. 4a–l). SEM examination showed the morphological type of fibroblasts on the chitosan film was in the first stage of the whole process of adhesion and spreading of cells. Moreover, the cell spreading on the chitosan film was less than the blend ones. It may be attributed to the lower surface energy and the existence of some inter-molecular interactions of chitosan functional groups. However, at the same culturing period, fibroblasts morphologies on Ch/PEGF blend films (80/20 and 60/40 in blend ratios) was in the attachment, filopodial growth, cytoplasmic webbing, flattening of the cell mass stages which is consistent with the situation expected for biocompatible materials.

As illustrated in Fig. 3b–j, the attached cell counts on the film with 80/20 in blend ratio is more than those adhered to the blend films with 60/40 and 40/60 blend composition ratios. It was published that the modified surfaces with PEG-base hydrogels allowed efficient control over cell adhesion. The contact angle of the blend with 80/20 in blend ratio was reported at 59.90°, showing that these materials had good hydrophilicity thereby they were suitable for cell supporting (Hashemi Doulabi et al. 2013b; Solouk et al. 2011). In fact, the simultaneous existence of Ch and PEGF on the surface could influence surface energy and surface charge; therefore, there are synergistic effects on the cell growth.

The amounts of cell spreading (µm^2^) and cell count (cell/mm^2^) on chitosan film and its blends with PEGF in different blend Ch/PEGF ratios after culturing for 1 day are summarized in Table 1. The accurate measurement of cell count and cell spreading was not possible on the third day of incubation. On day 5, the morphology of fibroblasts was in flattened and networking stages; however, the cell count measurement was not also possible due to the forming a single layer of fibroblasts. The cells could attach to the surface of chitosan film and Ch/PEGF blend films. In other words, the addition of the PEGF into chitosan for improving physicochemical and mechanical properties did not impair biological properties of chitosan. These films were biocompatible and provided a suitable substrate for fibroblasts growth. This finding could be confirmed by MTT assay in the next section. Similar to blends based on chitosan and PEG, the results showed that blends of Ch/PEGF could improve cell adhesion (Zhang et al. 2002). Adding PEGF up to 40% (by wt) could support cells; however, PEGF more than 60 wt% in the blend could decrease surface charge leading to reduced spreading and filopodia. The reason could be associated with the thickness and crystallinity of the films that could be confirmed with our previous finding (Hashemi Doulabi et al. 2013b; Uygun et al. 2010).

**Table 1:** The degree of fibroblasts cell spreading and the number of spreading cells on the blend films with various blend ratios and chitosan film after 1 day of incubation

Film composition	Chitosan	Ch/PEGF blend ratio		
		80/20	60/40	40/60
Cell area (m^2^)	464 ± 127	500 ± 71	496 ± 87	466 ± 107
Cell count (cell/mm^2^)	552 ± 110	530 ± 168	240 ± 130	65 ± 3

Thus, the film of Ch/PEGF with 80/20 in the blend ratio could be chosen as the best candidate for wound dressing material, which strongly supports our previous results (Hashemi Doulabi et al. 2013b).

### MTT assay

**Fig. 5:**
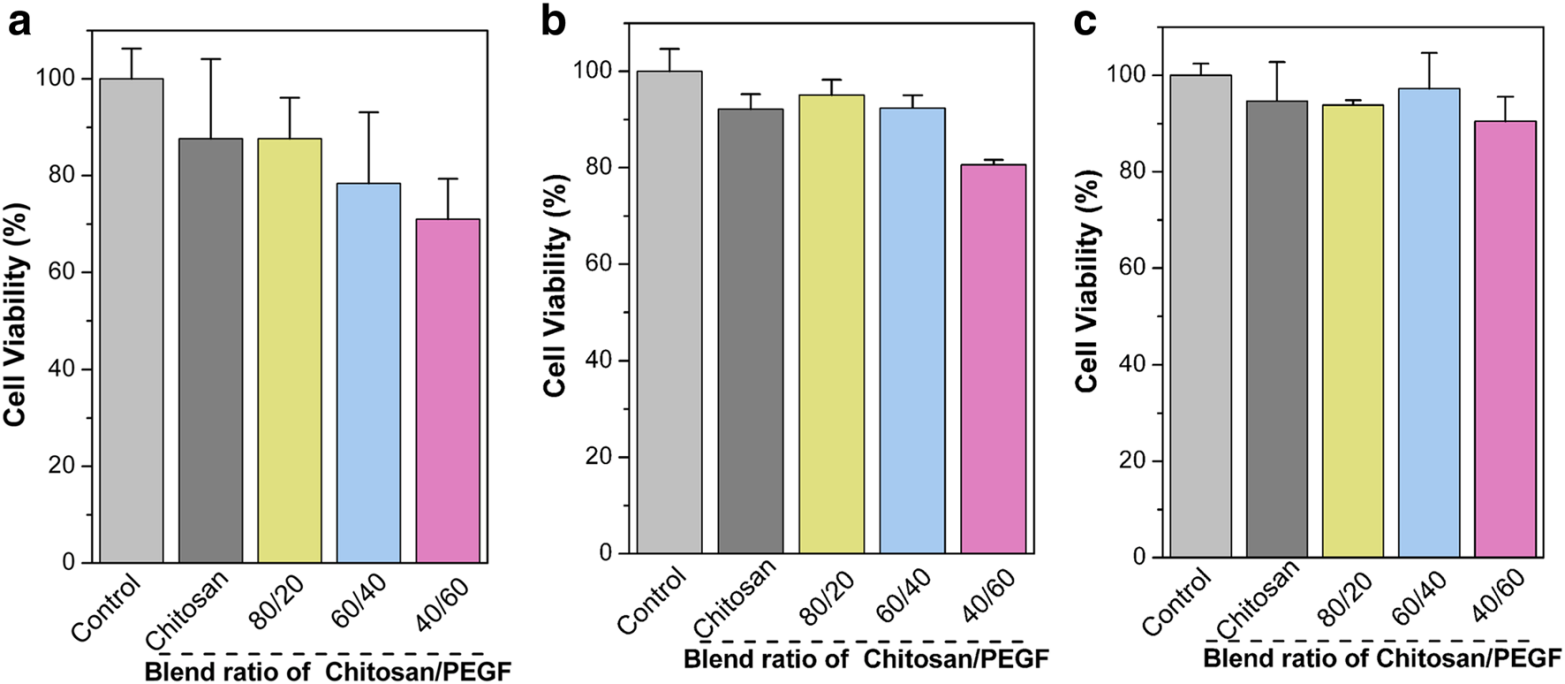
Cell viability results of Ch and Ch/PEGF blend films after 1 (**a**), 3 (**b**) and 5 (**c**) incubation days; tissue-culture polystyrene plate used as a control, *n *= 5

The MTT assay was accomplished to measure mitochondrial activity and cell viability. It relies on the ability of the viable cells to reduce a water-soluble yellow dye (MTT) to a water-insoluble purple formazan product. Figure 5a–c shows the cell viabilities of the Ch/PEGF blend films in contact with L929 fibroblasts after 1, 3 and 5 days of incubation, indicating these are non-toxic, biocompatible and cytocompatible (cell viability > 75%). According to Fig. 5, the blend ratio could significantly influence the cell viability at the 1st, 3rd and 5th days of incubation; the higher the Ch contents, the higher cell viability, for which SEM images confirmed MTT results. This may be due to the presence of amino groups in the Ch backbone. MTT results also showed that the blending process did not impair the cell viabilities of the blend films in comparison to the Ch film in the 1st, 3rd and 5th days of incubation (*p *> 0.05). As a final point, the cell behavior analyses highlight a good cell viability, proliferation, and distribution, pointing out a good biocompatibility profile of the Ch/PEGF films in contact with L929 fibroblast cells. In other words, Ch/PEGF with 80/20 in the blend ratio could promote cell attachment; therefore, chitosan could improve biological properties of PEGF.

## Conclusions

In this study, Ch/PEGF blend films were prepared with different blend ratios. In vitro degradation results revealed that any decrement in Ch/PEGF blend ratio resulted in a corresponding increase in their degradation rate due to higher PEGF water solubility. The pH evaluations made in the polymer degradation media showed the presence of that enzyme with a concentration similar to the human serum that was negligible. Cell behaviors toward all the formulations showed acceptable high biocompatibility and non-toxicity properties with fibroblast L929 cells under in vitro testing conditions. The film of Ch/PEGF with 80/20 of blend ratio was chosen as the optimum support for wound dressing applications due to biodegradation and biocompatibility properties as well as keeping their integrity during 28 days after incubation. We believe these films with such properties can be widely applied to biomedical applications such as a wound dressing material.
